# The impact of climate change on Korea’s agricultural sector under the national self-sufficiency policy

**DOI:** 10.1371/journal.pone.0313748

**Published:** 2025-01-24

**Authors:** Seulki Kim, Jiyong Eom, Ying Zhang, Stephanie Waldhoff

**Affiliations:** 1 Green Business and Policy Program, Korea Advanced Institute of Science and Technology, Daejeon, Republic of Korea; 2 School of Business & Technology Management, Korea Advanced Institute of Science and Technology, Daejeon, Republic of Korea; 3 Joint Global Change Research Institute, Pacific Northwest National Laboratory, Richland, WA, United States of America; University of Georgia, UNITED STATES OF AMERICA

## Abstract

Evolving environmental conditions due to climate change have brought about changes in agriculture, which is required for human life as both a source of food and income. International trade can act as a buffer against potential negative impacts of climate change on crop yields, but recent years have seen breakdowns in global trade, including export bans to improve domestic food security. For countries that rely heavily on imported food, governments may institute policies to protect their agricultural industry from changes in climate-induced crop yield changes and other countries’ potential trade restrictions. This study assesses the individual and combined effects of climate impacts and food self-sufficiency policies in Korea, which is highly dependent on imports. We use the Global Change Analysis Model (GCAM), a global integrated assessment model, to explore (1) the direct impact of climate change on Korea’s agricultural yields, (2) the full impacts of global climate change on agricultural production, including trade-induced changes due to yield changes in other regions, (3) the impacts of food self-sufficiency policy, and (4) the interactive impact of climate change and self-sufficiency policies. We find that, in Korea, the direct impact of climate change on agricultural yields would be overshadowed by the impact of global climate change due to changing trade patterns. Second, global climate change leads to a rise (rice and wheat) or a decline (soybeans) in Korean producer revenues, while simultaneously raising consumer expenditures on both staples and non-staples. Third, implementing self-sufficiency policies for wheat and soybeans in Korea boosts the nation’s producer revenues, in conjunction with the effects of climate change, at the cost of additional increases in consumer expenditures for both staples and non-staples.

## 1 Introduction

Among all sectors known to be exposed to global climate change, the agricultural sector is the most often cited due to the physical impacts of climate change on crops and arable land, and its economic consequences [1]. Previous studies on the impact of climate change on agriculture consistently show that it affects land use and crop yields directly through increased temperature and decreased precipitation [2–5]. The impact of climate change on agricultural yields affects food production, which could pose a risk to the global food supply, as revealed by key food security indicators, such as international and national food prices [1].

Studies report that international trade may play an important role in mitigating the impact of climate change on agriculture, such as channeling food to regions with domestic crop shortfalls [6]. However, there are signs that such trade is failing to work as a buffer, as exporting countries seek to meet domestic demand by reducing exports in times of reduced yields. In addition, borders may be closed due to factors like the global COVID-19 pandemic, resulting in agricultural exports that are stuck in ports and instability in supply and demand [7]. As a result, adopting a protectionist stance, several major crop-producing nations have established export restrictions [8].

Against this backdrop, countries have developed food security policies to alleviate possible market risks in the foreseeable future, with some researchers arguing that domestic agriculture can be the primary source of food supply for all people and a source of income for those employed in related industries [9]. Clearly, it is necessary to assess the impact of global climate change on domestic agriculture and, in light of new food security policies, how such policies can interact with the impacts of climate change on important outcomes such as food consumption.

Our study addresses the following two questions: (i) What will be the direct impact of climate change on agricultural production, imports and exports, producer revenues, consumer food expenditures, and food consumption in Korea in the future? (ii) What will be the full impacts of global climate change, including trade-induced impacts due to yield changes in the rest of the world? (iii) What will be the impacts of agricultural self-sufficiency policy on those outcomes? (iv) What will be the interactive impact of climate change and self-sufficiency policy?

We focus on the case of Korea, which relies heavily on imports for its domestic food supply and where global climate change is expected to have large impacts on the agricultural sector [10]. We quantify the impact of climate change on producer revenues for the top three food security-related crops in the country—rice, wheat, and soybeans—and the impact on consumer expenditures for staple and non-staple crops. We demonstrate that the impact of climate change on the agricultural sector can influence income stability for crop producers and threaten adequate food supply for consumers. In addition, we explicitly take global agricultural trade into account such that domestic and imported crops, both of which are subject to climate change impacts and compete with each other to occupy the domestic market. We examine the situation in both the absence and presence of Korea’s self-sufficiency policy for wheat and soybeans, which requires a minimum fraction of domestic consumption of wheat and soybeans to be satisfied by domestic production instead of imports.

Here, we employ the Global Change Analysis Model (GCAM), a global Integrated Assessment Model, which systematically links the economy, agriculture and land use, water, energy, and climate systems [11] under scenarios of climate change and self-sufficiency policy. We draw on a dataset of long-term, country-level, annual yield shocks for 12 crops projected by multiple global circulation models under the Representative Concentration Pathway of 8.5 W/m^2^ (RCP8.5) [5]. We apply these yield shocks as scenario inputs to GCAM and examine the outcomes from 2015 to 2050 in five-year time steps. In addition, we add constraints on domestic production and consumption of wheat and soybeans into the model to simulate the self-sufficiency policy imposed on the two crops in Korea [12].

Our contribution to the literature is twofold. First, to the best of our knowledge, our study is the first of its kind to perform an integrated assessment of climate change impact on a domestic agricultural sector with consideration of global trade patterns and a national self-sufficiency policy. Second, our analysis quantifies the impact on domestic crop production, imports and exports, crop producer revenues, consumer food expenditures, and food consumption, which are crucial for informing policymakers.

Our scenario results indicate that, first, in the case of Korea, the impact of global climate change significantly outweighs the domestic impact of changing climate in terms of crop production, consumption, producer revenues, and consumer expenditures. Second, we find that global climate change leads to an increase in revenues for rice and wheat producers in Korea, soybean producers experience a decrease in revenue, and consumer expenditures for both staple and non-staple food increase only slightly. Lastly, by implementing the self-sufficiency policy for wheat and soybeans in Korea, producer revenues rise, both with and without the impact of climate change. However, this may result in a small increase in consumer spending for both staples and non-staples.

The remainder of the paper is structured as follows. Section 2 explains the background of this study and describes the characteristics of the Korean agricultural system and its position in the international market. Subsequently, we review previous literature that analyzes the impact of climate change on agriculture from various perspectives. Section 3 describes the model used in this study, GCAM, particularly its agriculture and land use module, and the scenario configuration designed to answer the research questions. Section 4 discusses key results related to the individual and interactive impact of climate change and self-sufficiency policy on Korean agriculture from the viewpoints of producers and consumers based on the model outputs. Concluding remarks are presented in Section 5.

## 2 Background

### 2.1 An overview of the agricultural sector in Korea

Domestic agriculture is a vital source of income and secure food supply in the Republic of Korea. Although the proportion of agricultural employment in Korea to total employment continues to decline, 4.5% of Koreans are agricultural producers and 16% of the total land area is devoted to agricultural land use [13]. Domestic producers’ income from growing food crops account for more than 20% of farm household income [14]. Among food crops, rice production accounts for the highest proportion at 16.2%; the production of other grains is low in the limited cultivated area. While rice production in Korea is sufficient to meet consumers’ demand, production of wheat and soybeans, which are the other two most highly consumed crops, is very low compared to domestic demand.

Korea’s agricultural structure has changed over time, with increased dependence on trade. As domestic agricultural production is insufficient to supply food for the whole country, there is a high dependence on international trade to balance the supply and demand for diverse food crops. Since the Uruguay Round Agreement on Agriculture in 1994, which marked the beginning of trade liberalization, imports in the Korean agricultural sector have doubled [15]. Korea’s food self-sufficiency ratio (SSR), which is defined as the percentage of food consumed that is produced domestically [16], decreased from 50.8% in 2016 to 45.8% in 2019; that is, more than half of the grains consumed as food in Korea were imported from abroad.

The vulnerability of agriculture in Korea to trade-related shocks is high because of the nation’s significant reliance on trade. According to the recent evaluation of the Korean food supply system conducted by the Korean government [17], Korea imports most of its major grains; therefore, international market conditions are important for the domestic market. For example, the domestic market is subject to changes in global crop prices due to market disturbances such as supply chain failures during the pandemic. Similarly, if climate change reduces crop production worldwide and crop exports are restricted in major exporters, national food security could become a serious problem for countries that rely heavily on imports, like Korea.

In response to climate change, the Korean government is taking measures to improve food security and protect the Korean agricultural sector from unexpected changes in the global market. First, with the announcement of the Second Basic Plan for Climate Change Response [17], the government pointed out that climate change could threaten food security and highlighted the need for self-sufficiency measures. Also, in preparation for a future agricultural crisis, the Korean government announced the National Food Plan, a comprehensive response to various food security problems related to the environment, health, and safety. Target crops in the plan include rice, wheat, and soybeans, which have the highest domestic consumption. In particular, the plan focuses on increasing the self-sufficiency levels of wheat and soybeans, crops for which a very high proportion is imported from overseas. Thus, it is important to understand the impact of climate change on agriculture in light of global trade and to evaluate the suitability of new policies in the climate crisis era.

For our analysis, we choose Korea’s top three most heavily consumed crops: rice, wheat, and soybeans [18]. The recently announced National Food Plan [12] also places importance on rice, wheat, and soybeans in terms of food security.

### 2.2 Literature on the impact of climate change on the agricultural sector

Calvin et al. [19] outline four main pathways that link crop yield changes to economic welfare, through market outcomes in the agricultural sector. These pathways trace the progression from human activities to climate change, including shifts in carbon dioxide concentration levels, resultant crop yield changes, and market outcomes—including alterations in cropland area, production, consumption, and prices—culminating in impacts on economic welfare. Initial research by Zhao et al. [20] evaluated crop yield responses to the biophysical effects of climate change. Subsequent studies expanded this by investigating the impact on the economic sector, taking into account commodity prices, supply, and demand [21–27].

Another line of literature underscores the significance of considering international trade when evaluating the overall impact of climate change on agriculture. Baker et al. [21] suggest that national assessments of climate change impacts on agriculture could be skewed if the influences of global market dynamics and future productivity assumptions are not included. Zhang et al. [28] highlight that, via trade, the impact of climate change on the rest of the world could substantially alter domestic agricultural production. Snyder et al. [29] also highlight the necessity of accounting for the indirect effects emanating from international markets on regional agricultural impacts.

The prevailing insight from earlier studies is that international trade serves as a buffer to the impact of climate change on regional agriculture. Baldos and Hertel [6] found that international trade has the potential to alleviate the long-term impact of climate change on food security. Gouel and Laborde [30] indicated that changes in trade flows due to climate-induced changes in yields and large price movements can lead to economic welfare changes. However, this line of literature focused exclusively on the role of international trade, omitting the influence of country-specific agricultural policies.

Concerns over domestic agriculture’s vulnerability to climate change and global market disruptions have prompted a trend towards protective national agricultural measures, challenging the effectiveness of trade as an adaptive strategy for climate-related agricultural shifts. However, the majority of research to date has focused on the agricultural sector’s response to national or regional greenhouse gas (GHG) mitigation policies. Examples include Hasegawa et al. [24], which introduced a model that combines the effects of climate change with GHG reduction efforts to assess the interplay between climate policy and food security. Van Meijl et al. [31] examined the economic consequences of GHG mitigation measures on the agricultural sector, and Havlík et al. [32] crafted scenarios to assess how shifts in the livestock sector, influenced by various mitigation policies, could affect food availability. Despite these efforts, there remains a significant research gap regarding the influence of protective agricultural measures on food security.

Our study aims to fill this void by examining the impacts of climate change on agriculture, specifically focusing on the role of national food self-sufficiency policies. We delve into the effects of such a policy in Korea, assessing its impact on the agriculture sector and its repercussions for food production and consumption within the country. One related climate impact study is Kwon et al. [33], which indicated that climate change in Korea could alter agricultural productivity and prices, potentially affecting the nation’s GDP. Our research builds on this, contextualized within Korea’s self-sufficiency policy, examining a broad spectrum of market outcomes influenced by climate change, such as domestic crop production, trade, farmers’ revenues, consumer food spending, and overall food consumption.

## 3 Methods

### 3.1 The global change analysis model

Climate change directly impacts regional agriculture production through changes in temperatures, precipitation, and other climatic factors. However, there are also indirect effects, through changes in yields in other regions, impacting trade and prices, and connections with the energy (demand for bioenergy) and water systems (water supply for irrigation). To improve our understanding of the full suite of climate change impacts on agriculture, we use a global Integrated Assessment model, the Global Change Analysis Model (GCAM) v5.4. GCAM is an internally consistent, fully integrated model of the coupled human-Earth system, encompassing energy, water, land, socioeconomics, and climate. GCAM can be used to assess the impacts of economic and environmental policies [34]. Within GCAM, the world is divided into 32 geopolitical regions within the energy system, 235 water basins within the water system, and 384 global land units (GLUs) within the land system. GCAM’s calibration is based on the historical reference year of 2015, and it projects essential variables into the future up to 2100 with 5-year model periods. In this study, we track the outcomes spanning a period of 2015 to 2050. For more details, please refer to the model documentation available at http://jgcri.github.io/gcam-doc/v5.4/toc.html.

The agriculture sector in GCAM derives market outcomes from data related to the supply of, demand for, and trade of crops. The regional supply of crops consists of domestic and imported crop production. GCAM models four types of agricultural management practices: the combination of (i) rain-fed versus irrigated and (ii) high versus low fertilizer application. These management types have different yields, costs, and profit rates. The model endogenously determines crop production in every region, including management type and associated land allocations, based on a logit choice algorithm that takes into account profit rate differences among crops and management types [35]. In essence, crop production changes through either the expansion of cultivated land (extensification) or advancements in agricultural technology (intensification). Domestic crop production can be consumed domestically or exported to the global market.

**Fig 1:**
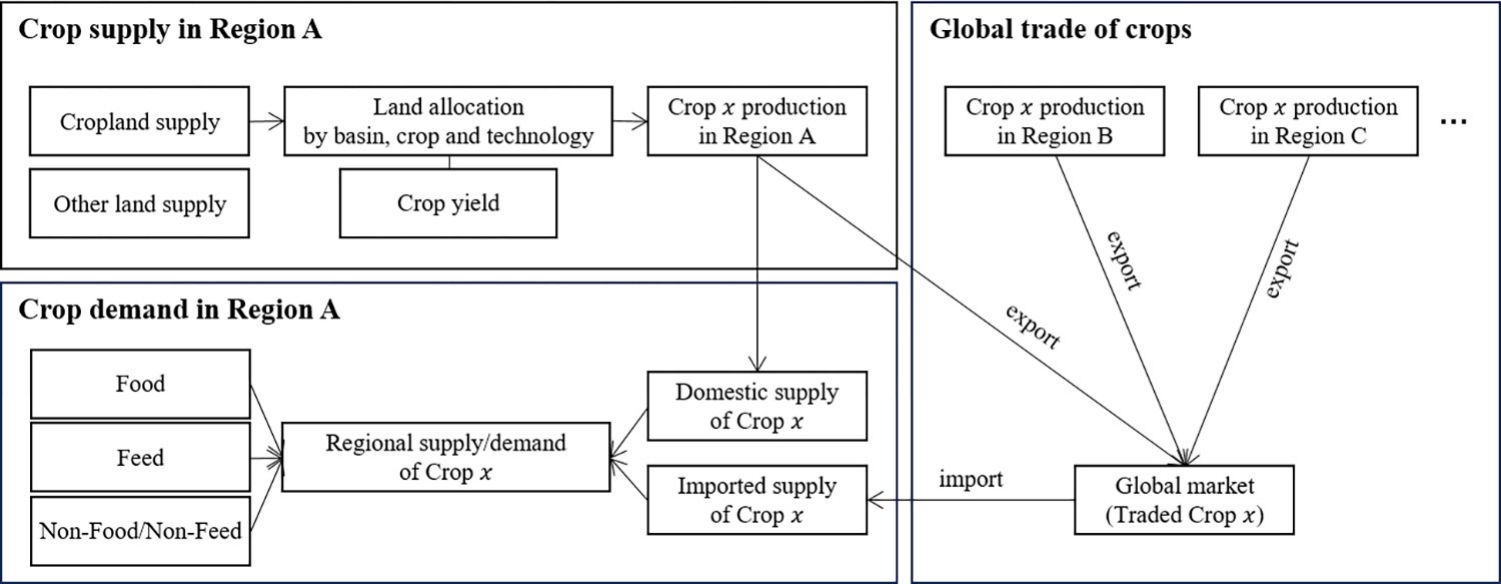
GCAM structure related to agricultural market.

GCAM is a market equilibrium model, so the demand for a commodity in a regional market equals to the supply of the commodity in the regional market (Fig 1). The total demand for a crop consists of the food demand, feed demand, and non-food non-feed demand. Total regional food demand is determined by population and per capita demand for foods, which in turn is a function of per capita income, and commodity prices [36]. GCAM assumes population and income growth based on a modified version of the Shared Socioeconomic Pathway 2 (SSP2) that better reflects historical changes in population and GDP. The resulting demand (referred to as regional crop) is met by crops from both the regional and global markets. The model of food demand used in GCAM has two main categories: staples and non-staples. Staples are aggregated goods composed of grains and roots and tubers, while non-staples include all other food commodities (e.g., animal products, sugar, oils, fruits, and vegetables). The classification of GCAM’s agricultural commodities into staples and non-staples can be found in the GCAM documentation [34]. For the crops of interest in this study, rice and wheat are classified as staples, and soybeans are classified as non-staples.

As supply and demand shift in the future, GCAM endogenously solves for the market equilibrium prices of all modeled commodities, including agricultural commodities, such that supply equals demand for each commodity [19]. The GCAM follows the convention of FAO in defining agricultural commodity prices, that is, prices that producers receive from the market for primary crops. Consumers purchase commodities in the regional market that is supplied by domestic production and imports, and pay regional prices that are determined by the weighted average of domestic producer prices and global import prices. Overall prices of food consumption—staples and non-staples (Table 1)—are determined by the food demand model [36]. Consumers’ price and income elasticities change with income level, such that demand is more elastic at low-income levels than at higher-income levels for both staples and non-staples.

**Table 1:** Classification of staples and non-staples in the GCAM.

Staples	Non-staples	
Corn	Beef	Soybeans
OtherGrain	Dairy	PalmFruit
Rice	MiscCrop	Pork
RootTuber	FiberCrop	Poultry
Wheat	OilCrop	SheepGoat
	SugarCrop	OtherMeat_Fish

GCAM represents international trade in agricultural products using a modified Armington model, with goods from different regions assumed to be imperfect substitutes. In the model, regional exports and imports are linked to one global trade pool, where prices for domestically produced commodities are regionally differentiated for each crop, and trade flow is tracked [37, 38]. The model captures the common situation in the world of agriculture that the impact on agriculture in one country spreads to others through trade.

### 3.2 Scenario configuration and development

We assess the impact of climate change on Korea’s agriculture sector from the perspectives of both producers and consumers. The impact on the producer side is evaluated as production, producer prices, and producer revenues (production multiplied by producer prices). The impact on the consumer side is evaluated as consumer prices, consumption, and consumer expenditures (consumption multiplied by consumer price).

We build climate change scenarios based on the yield shocks from Waldhoff et al. [5]. The study sources of monthly average temperature and total precipitation data from the three Earth system models or General Circulation Models (GCMs)—the Community Climate System Model (CCSM4), Geophysical Fluid Dynamics Laboratory (GFDL), and Hadley Centre Global Environment Model (HadGEM_ES)—to represent hotter/drier, moderate, and cooler/wetter future projections. Taking into account temperature and precipitation specific to each region, the estimated yield shocks from the study are used to generate country-level annual yield trajectories for each ESM’s realization of the Representative Concentration Pathway 8.5 (RCP 8.5). To incorporate the climate change impact on crop yield in GCAM, we map the crops in Waldhoff et al. [5] to GCAM commodities and apply the projected yield shocks described above to each of the four different agricultural management types.

To implement the self-sufficiency policy scenario in accordance with the National Food Plan announced by the Korean government, we constrain the self-sufficiency ratio (SSR; Eq 1) of wheat to be at least 5% and that of soybeans to be at least 33%, with no change in SSR for rice, beginning in 2025. We implement the constraints in GCAM by creating a market for the self-sufficiency credit associated with each crop’s domestic production [39]. For each unit of a crop produced by a domestic producer, one unit of self-sufficiency credit for this crop is created. Consumers are forced to pay a proportion of the unit credit when purchasing one unit of crops in the regional market. The credit paid by consumers goes to domestic producers in the form of subsidies, stimulating domestic production.


$$SSR=\frac{Production}{Production+Imports-Exports}=\frac{Production}{Consumption}$$ Eq. 1

In order to assess the impact of climate change on domestic agriculture sector and the possible confounding effects of agricultural self-sufficiency policies, we develop five scenarios (Table 2). The first scenario is the reference case without climate change or a self-sufficiency policy (“Ref”). The second reflects the domestic impact of climate change only (“Ref_dCC”) assuming no climate change in the rest of the world. The third represents the full global impact of climate change (“Ref_gCC”), where the impact of climate change in the rest of the world can affect domestic market outcomes through international trade. We then compare Ref and Ref_dCC to assess the direct impact of climate change on domestic crop production, and Ref and Ref_gCC to evaluate both how climate change impacts domestic crop production and how the impact of climate change on global crop production influences domestic production through trade.

**Table 2:** Scenarios.

Scenario name	Climate change location	Policy
Ref	No climate change	No additional policy
Ref_dCC	Only in Korea	No additional policy
Ref_gCC	Global	No additional policy
SS	No climate change	Self-sufficiency policy on wheat and soybeans
SS_gCC	Global	Self-sufficiency policy on wheat and soybeans

Another set of scenarios applies the self-sufficiency policy to wheat and soybeans, including the policy scenario without climate change (“SS”) and with the full global impact (“SS_gCC”). Note that the no-policy scenarios (“Ref,” “Ref_dCC,” and “Ref_gCC”) are reflective of current trade preferences and trade-related measures for individual GCAM regions, including Korea. We compare Ref and SS to assess the impact of the self-sufficiency policy on wheat and soybeans on trade patterns, and Ref_gCC and SS_gCC to assess the impact of the policy on trade patterns in the presence of global climate change.

We also evaluate the impact of climate change with and without the self-sufficiency policy. To compare the effects, we analyze the difference between SS_gCC and SS, which represents the full impact of climate change with the policy. Similarly, we find the difference between Ref_gCC and Ref, representing the full impact of climate change without the policy. We then compare the two differences to evaluate the extent to which the impact of climate change is alleviated or amplified by the policy based on various outcomes of production, consumption, producer revenues, and consumer expenditures.

## 4 Results

### 4.1 The impact of climate change on producers and consumers

**Fig 2:**
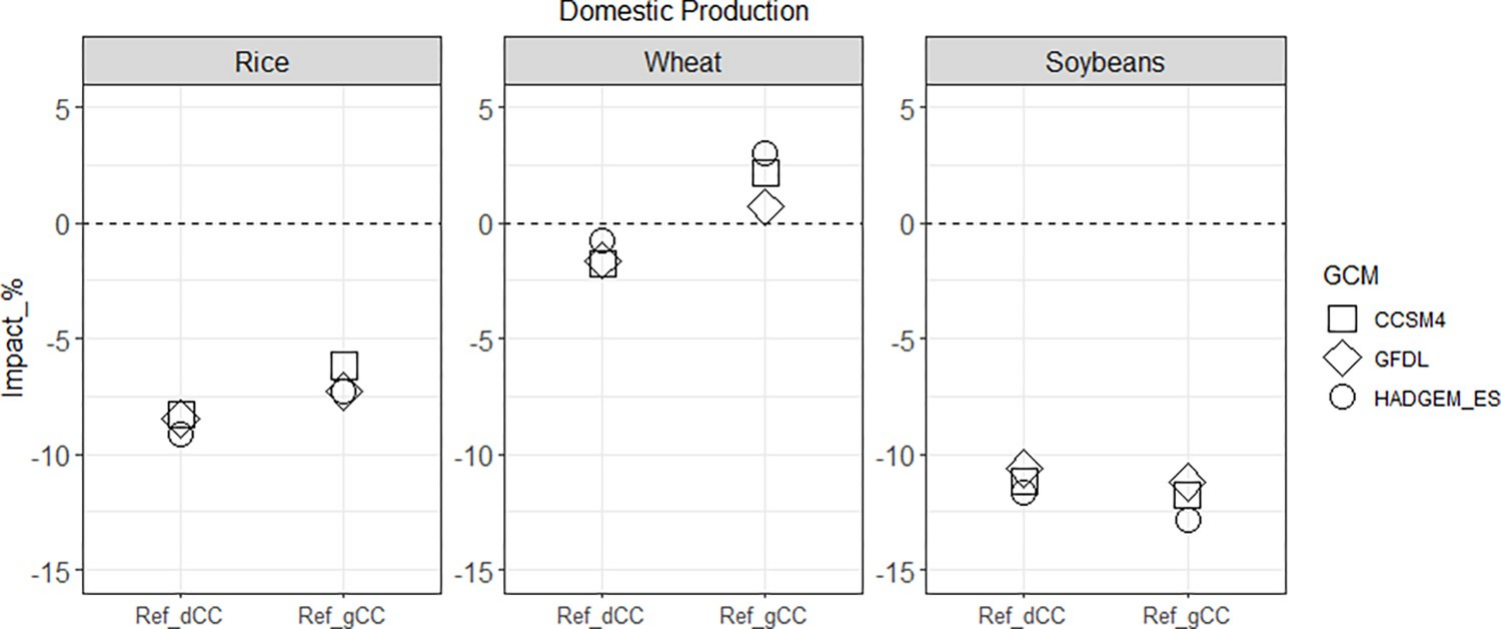
Changes in Korea’s domestic production of rice, wheat, and soybeans by 2050: Percentage differences compared to the Ref scenario for both Ref_dCC and Ref_gCC cases.

**Fig 3:**
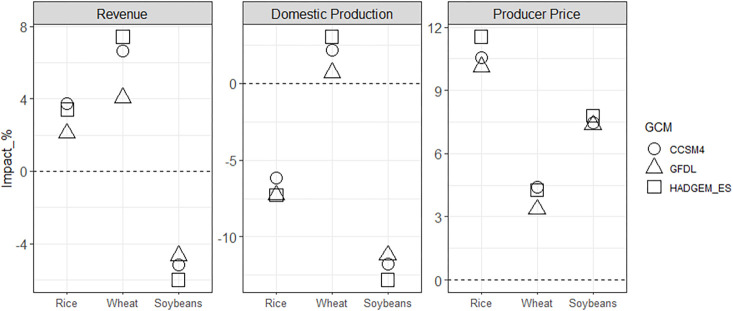
Changes in Korea’s producer revenues, domestic production, and producer prices of rice, wheat, and soybeans by 2050: Percentage differences compared to the Ref scenario for Ref_gCC case. Note that the scales on the y-axis vary for each category.

**Fig 4:**
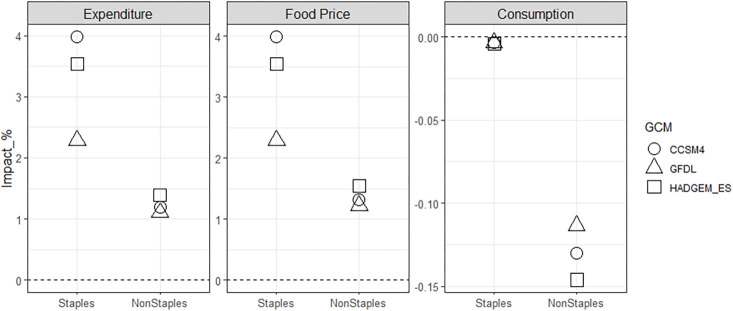
Changes in Korea’s consumer food expenditures, food prices, and food consumption of staples and non-staples by 2050: Percentage differences compared to the Ref scenario for Ref_gCC case. Note that the scales on the y-axis vary for each category.

Three notable results for Ref_dCC and Ref_gCC arise in our scenario assessment. First, the impacts of global climate change on domestic crop production in Korea vary depending on the level of import dependency (Fig 2). Second, the impact of climate change on producers varies depending on the crop (Fig 3). Lastly, changes in production and price due to climate change result in higher consumer spending on staples than non-staples (Fig 4).

We see different impacts on the domestic production of crops between Ref_dCC (climate change impacts on domestic crop yields only) and Ref_gCC (climate change impacts on global crop yields) (Fig 2). Rice and soybean production drop significantly and wheat production shows a relatively small decrease under Ref_dCC relative to Ref (no climate change impacts on crop yields). In Ref_gCC, rice production is also lower than Ref, although the effect is slightly mitigated relative to Ref_dCC. Wheat production with Ref_gCC is increased compared to both Ref_dCC and Ref. Compared to rice and wheat, the difference in soybean production is relatively small between Ref_dCC and Ref_gCC, with both showing large declines (~10–12%) relative to Ref.

The relative results of Ref_dCC and Ref_gCC demonstrate the domestic crop production changes due to climate change impacts on crop yield in the rest of the world. The yield changes in Korea and elsewhere lead to changing comparative advantages in crops across regions, which result in changes in crop trade patterns. Recognizing the importance of this, the remainder of our analysis assesses the impact of climate change on crop yields globally.

The result aligns with the findings of Mosnier et al. [40] and Lee et al. [41]. Mosnier et al. emphasized that domestic climate change impacts can be overshadowed by global trade effects, which is consistent with the shifts in crop trade patterns observed in our results. Additionally, Lee et al. highlighted how climate change and global trade dynamics contribute to rising food costs for net-importing countries like South Korea, paralleling our finding that consumer spending increases on staples due to climate change. These similarities reinforce the importance of considering both domestic and global factors in understanding the full scope of climate change’s impact on agricultural production and trade.

Fig 3 shows that the changing comparative advantage of Korean production of rice, wheat, and soybeans under global climate change results in decreased production for rice and soybeans (~7% and 12%, respectively) and increased production for wheat (~2%). The producer prices for these crops all rise in Ref_gCC relative to Ref, though the magnitudes vary by a factor of 3. The combination of changing prices and production leads to very different producer revenue outcomes for each crop. For rice, the increase in price offsets the decrease in production, resulting in higher revenue than Ref. Because both the price and production increase for wheat, the revenue also increases. However, the relatively moderate increase in producer price for soybeans does not sufficiently offset the relatively large decrease in production, leading to a net decrease in producer revenue.

**Fig 5:**
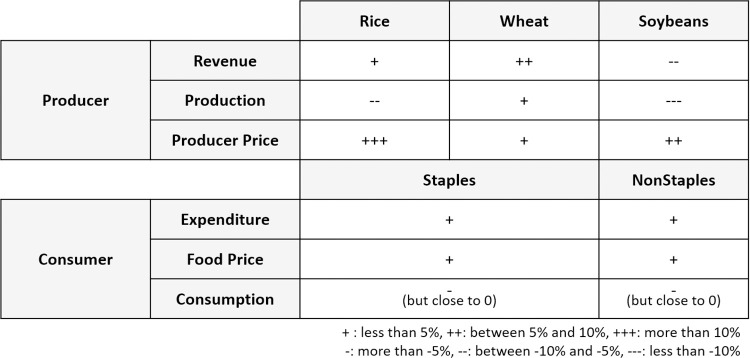
2050 climate change impacts on producers and consumers: A comparison of the Ref_gCC and Ref scenarios, with results averaged across General Circulation Models (GCMs).

Changes in each crop’s domestic producer price and global (import) price affect the price in the regional market (i.e., Korea), which leads to changes in food prices. The changes in food consumption are much smaller than the changes in food price, as food demands, particularly staple foods, are highly price inelastic. The combined effects of the large price changes result in higher consumer food expenditures, especially on staples (grains, roots and tubers), which is composed of about 40% rice and wheat, and the percentage increase in consumer expenditures closely mirrors the pattern of price changes of both staple and non-staple food. Fig 5 provides a qualitative summary of the impacts of climate change on domestic producers and consumers.

### 4.2 The impact of self-sufficiency policy on producers and consumers

**Fig 6:**
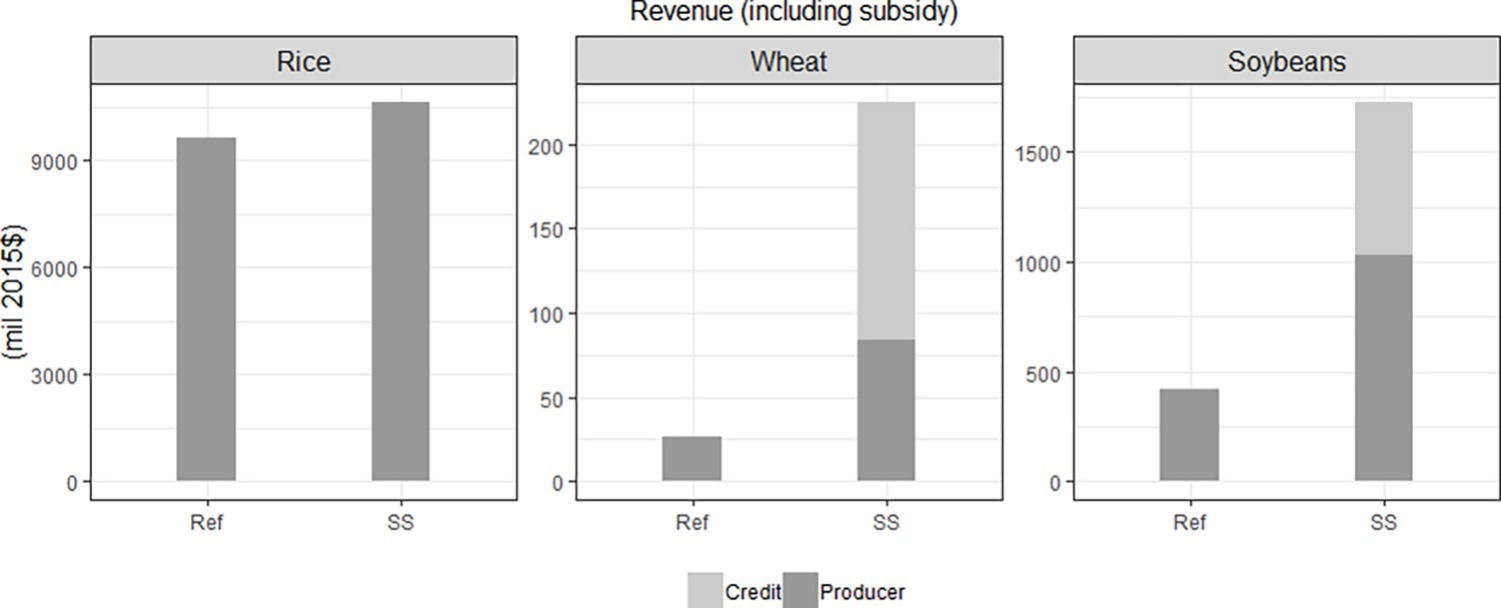
2050 producer revenues with subsidies in Korea: A comparison between the Ref and SS scenarios for rice, wheat, and soybeans. Note that “Credit” indicates revenue from the self-sufficiency subsidies, while “Producer” refers to the revenue obtained from domestic sales at the domestic producer price, excluding subsidies. The scales on the y-axis differ for each category.

**Fig 7:**
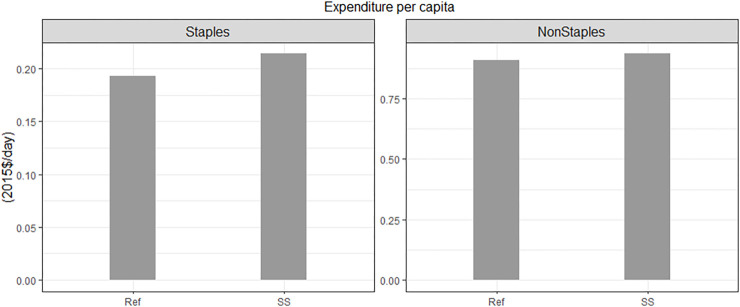
2050 per capita consumer food expenditures in Korea: Results from the Ref and SS scenarios, comparing expenditures on staple and non-staple foods. Note that the scales on the y-axis vary for each category.

In this section, we examine the impact of self-sufficiency policy on producers and consumers without climate change. Our self-sufficiency (SS) policy functions by incentivizing increased domestic production through subsidies, resulting in heightened producer revenues when compared to the Ref (Fig 6). Nevertheless, the rise in consumer expenditures on food remains relatively modest (Fig 7), as food consumers bear only a partial cost of these subsidies through higher food prices. Notably, the implementation of the self-sufficiency policy leads to a substantial surge in wheat and soybean production, given that their domestic production under Ref falls below the policy’s prescribed threshold. In contrast, rice production remains unaffected, as it is not restricted by our policy scenario. Importantly, the increased competition for cropland due to expanded wheat and soybean production results in higher rice prices, consequently boosting revenue for rice production.

### 4.3 Interaction between climate change impacts and self-sufficiency policy on producers and consumers

**Fig 8:**
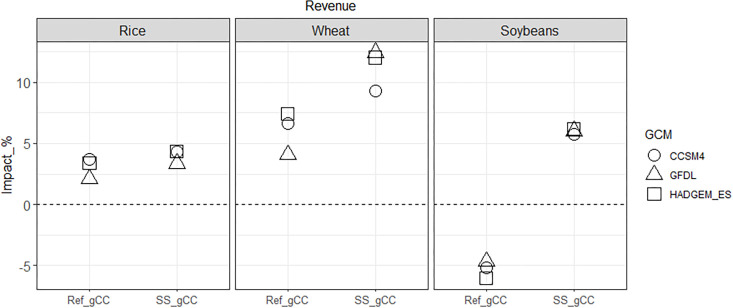
2050 producer revenue changes in Korea for rice, wheat and soybeans: Percentage impacts of the ‘Ref_gCC’ scenario (compared to Ref) and the ‘SS_gCC’ scenario (compared to SS). Note that the former shows the impact of climate change when the self-sufficiency policy is absent, and the latter shows the impact of climate change when the self-sufficiency policy is in place.

In this section, we examine the combined effect of climate change and the self-sufficiency policy to explore how the policy might moderate or augment the impact of climate change on producers and consumers. The climate change impact when the policy is in place (SS_gCC) results in increased producer revenues relative to SS for all crops in 2050. However, the climate change impact when the policy is absent (Ref_gCC) results in increased producer revenues for rice and wheat and decreased producer revenue for soybeans in 2050, compared to the Ref case. As a result, the differences comparing the two effects are particularly strong for soybeans, followed by wheat (Fig 8). The additional revenue from subsidies under the self-sufficiency policy for wheat and soybeans incentivizes producers to further expand land and intensify agricultural inputs to cultivate crops in response to the impact of climate change on crop yields.

**Fig 9:**
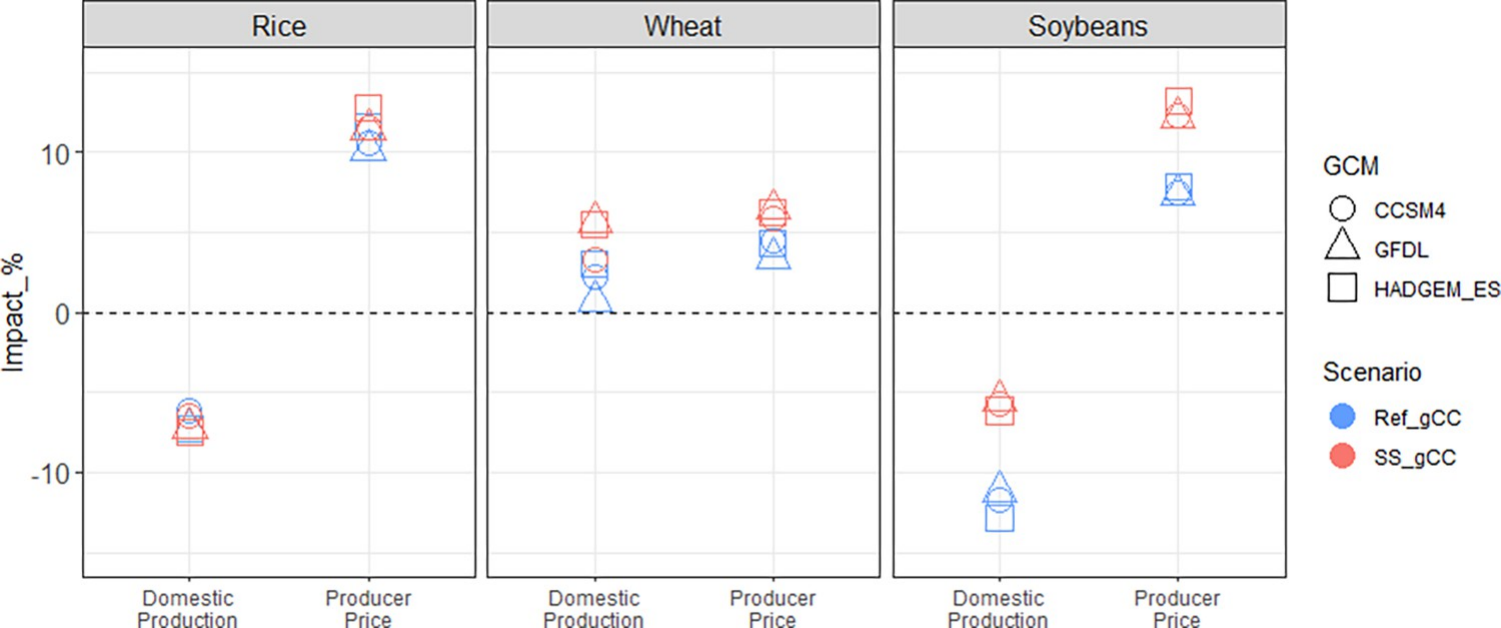
2050 domestic production and producer price changes in Korea for rice, wheat, and soybeans: Percentage impacts of the ‘Ref_gCC’ scenario (compared to Ref) and the ‘SS_gCC’ scenario (compared to SS). Note that the former shows the impact of climate change when the self-sufficiency policy is absent, and the latter shows the impact of climate change when the self-sufficiency policy is in place.

Now, the changes in producer revenue are dissected into changes in domestic production and producer prices. The adaptive response of domestic wheat and soybean production to climate change is augmented under the self-sufficiency policy; that is, the negative impact of climate change (if any) is alleviated with the policy in place. For example, the decrease in domestic production of soybeans due to climate change is less with the policy than without (Fig 9). Climate change leads to increased wheat production by a small amount, and the self-sufficiency policy motivates producers to produce wheat more aggressively. As a result, wheat production increases further, and soybean production decreases less, while the ripple effects on rice production are minimal as rice is not a target crop under the self-sufficiency policy.

The impact of climate change on domestic producer prices is augmented by the self-sufficiency policy (Fig 9). All three crops have greater increases in domestic producer prices when the policy is in place, most noticeably for soybeans. This is because the impact of 33% self-sufficient restriction on soybeans is larger than the impact of 5% restriction on wheat. Although rice is not directly subject to self-sufficiency restrictions and its production varies little when the policy is in place (Fig 9), the domestic producer price of rice still fluctuates due to land competitions and changes in demand.

**Fig 10:**
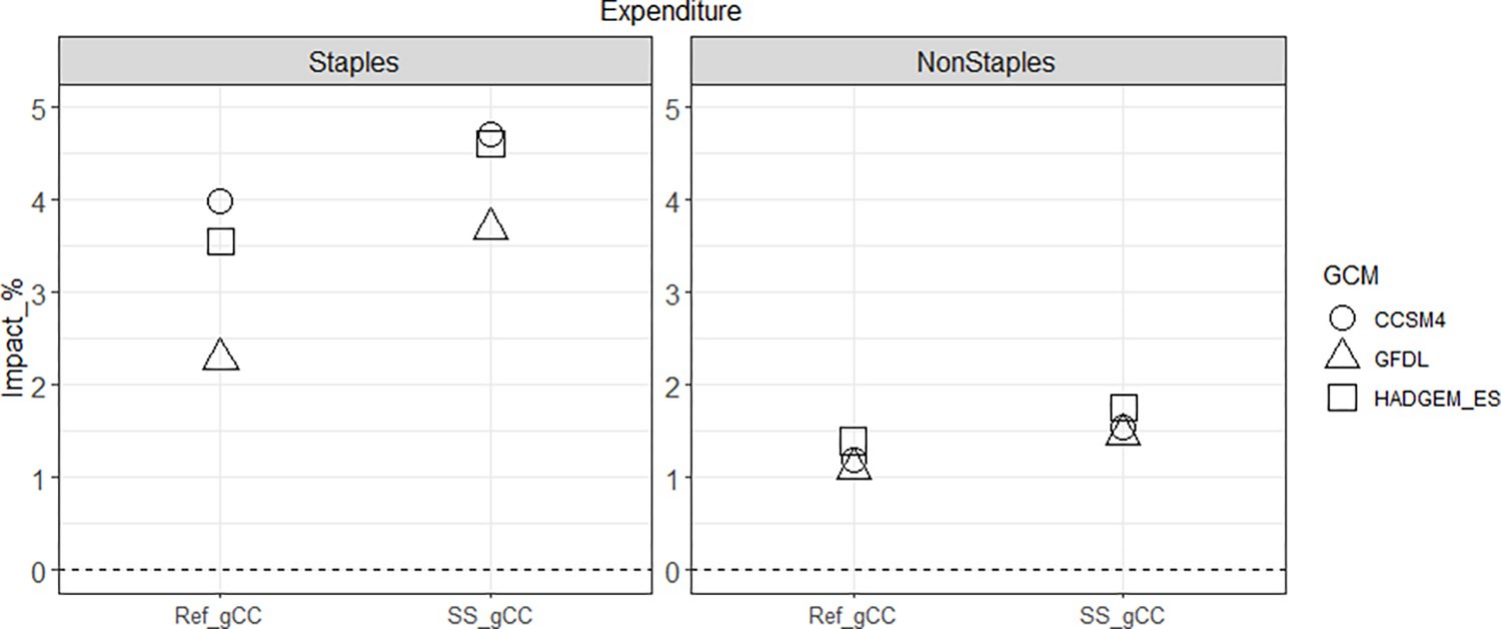
2050 consumer food expenditure changes in Korea for staples and non-staples: Percentage impacts of the ‘Ref_gCC’ scenario (compared to Ref) and the ‘SS_gCC’ scenario (compared to SS). Note that the former shows the impact of climate change when the self-sufficiency policy is absent, and the latter shows the impact of climate change when the self-sufficiency policy is in place.

On the other hand, climate change leads to modest increases in consumer expenditures on food when the policy is in place compared to the situation when the policy is absent (Fig 10). The effects are larger for staples than non-staples, attributed to their respective consumer prices and the corresponding demand.

**Fig 11:**
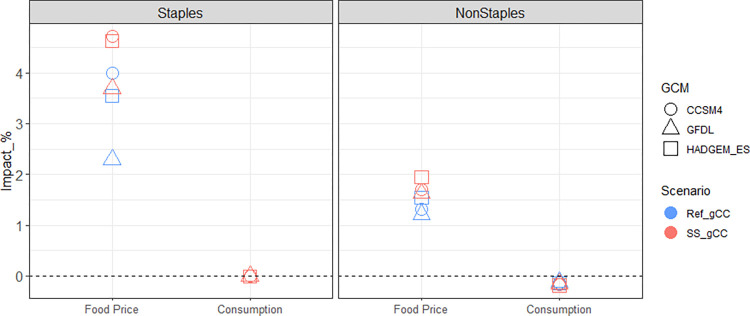
2050 food price and consumption changes in Korea for staples and non-staples: Percentage impacts of the ‘Ref_gCC’ scenario (compared to Ref) and the ‘SS_gCC’ scenario (compared to SS). Note that the former shows the impact of climate change when the self-sufficiency policy is absent, and the latter shows the impact of climate change when the self-sufficiency policy is in place.

The different changes in consumer expenditures can be attributed to different changes in food price and consumption. When the self-sufficiency policy interacts with climate change, the increase in the consumer price is larger than the increase in the consumer price when the self-sufficiency policy is absent, and both increases are larger for staples than non-staples (Fig 11). This is because the increase in the consumer price of staples reflects the rise in the domestic producer prices of rice and wheat, which consist of 88% of the staple caloric consumption, while the consumer price of non-staples is affected by the surge in the domestic producer price of soybeans to a lesser extent, as soybeans make up only 18% of non-staple caloric consumption. The indirect impacts of the self-sufficiency policy on other crops through cropland allocation and demand competitions, are relatively small. Similarly, the impacts on the consumer prices of non-staple animal commodities through changes in prices of feed crops is also small. The modest increase in the consumer prices of staples and non-staples has almost no impact on their consumption (Fig 11).

Zhang et al. [39] examined the changes in trade and cereal consumption under the assumption that all regions achieve a zero import dependency ratio, meaning 100% self-sufficiency for all regions, in the context of climate change. Their study found that climate change leads to higher cereal prices and decreased consumption, while import dependency constraints exacerbate the situation in regions with higher import levels. In our study, however, we applied the self-sufficiency targets set by the Korean government to assess the impact of climate change on market factors. We found that the increase in cereal consumption prices for consumers aligns with the results of Zhang et al.

## 5 Conclusions

The impacts of climate change on domestic crop yields are set to influence agricultural production, prices, revenues, consumer food consumption and expenditures. These outcomes are also susceptible to external shocks transmitted through various channels, including climate change impacts on crop yields elsewhere and shifts in domestic agricultural policy. Global agricultural trade has been an indispensable factor in maintaining global food security and has potential as a buffer against climate-induced damages. Nonetheless, depending on their market position, governments may opt to limit exports to safeguard the welfare of their citizens.

This study contributes to the literature by investigating the effect of climate-related physical risk on Korea’s agricultural outputs and the ensuing economic impact on producers and consumers, in terms of prices, revenues, and expenditures. It explored global trade dynamics and the interplay between climate change impact and a policy aimed at agricultural self-sufficiency. An integrated assessment model, incorporating agriculture and land use, was utilized to evaluate the impact of global climate change on domestic agriculture, taking into account the new self-sufficiency policy. The study offers valuable perspectives for assessing the impacts of climate change and devising strategic agricultural trade policies to address these challenges.

First, the study highlights the importance of considering trade as a conduit through which changes in the global agricultural environment are communicated. Korea’s agriculture is greatly impacted by the international market due to its heavy reliance on imports of rice, wheat, and soybeans. The findings indicate that focusing solely on the domestic climate impact, without considering the global impact leads to significant discrepancies. This is because domestic agriculture is indirectly influenced through trade channels that convey the global impact of climate change.

Second, it is crucial to consider the impact of climate change on different stakeholders when assessing its effects on agriculture. Our results indicate that the impact of climate change on producer revenues varies across crop types. While rice and soybean production declines significantly, wheat production experiences a slight increase. Producer prices rise for all three crops. On the consumer side, higher producer prices of rice and wheat (staple) as well as soybeans (non-staple) lead to increased food prices, though there is little change in caloric consumption. In addition, climate change moderately raised consumer expenditures on both staple and non-staple food.

Third, it is of practical importance to consider the interplay between climate change impact and national agricultural policies. We find that implementing a self-sufficiency policy to boost domestic production of wheat and soybeans in Korea enhances the positive effects of climate change on producer revenues. This policy may mitigate the decline in crop production under climate change, as observed in the case of soybeans in our experiment. Producer prices of wheat and soybeans increase under the self-sufficiency policy alongside the production changes. Consumers pay slightly more for food than before the policy’s adoption, with little change in food consumption.

Given that the self-sufficiency policy aims to ensure food security, however, crop production is not the only factor we should consider. It is because food security is multi-dimensional including availability, accessibility, utilization, and stability [42]. The results indicate that Korea’s self-sufficiency policy increases production with only moderate consumer cost increases. This highlights the need for balance between incentivizing producers and protecting consumers, especially vulnerable groups. Ensuring the financial feasibility of these policies is also critical. Supporting producers while managing government spending is key to creating sustainable and effective solutions. Policymakers must carefully balance increasing production, keeping food affordable, and managing government costs for long-term.

While this work makes a valuable contribution, there are several caveats that may not capture all the nuances of Korea’s agricultural sector and global trade dynamics. First, in GCAM, trade is modeled through a central global market, where all regions export to and import from a single global trade system rather than through bilateral trade. While this approach may limit the model’s ability to precisely reflect the real-world trade flows between individual countries, it offers greater flexibility in adapting to evolving trade patterns over time. Consequently, this design accommodates shifts in trade dynamics that would not necessarily adhere to fixed patterns as conditions and policies change. Second, when applying policies related to national food security, this study focuses solely on Korea’s self-sufficiency policy regarding three crops, without explicitly considering other agricultural policies in Korea or those in other countries. For instance, the Korean government employs tariff measures, such as higher rates on imported rice, to protect domestic agriculture. Additionally, there are regulations that establish quality standards to raise the barriers for imports. In this context, we do not take into account Korea’s other agricultural policies. From a global perspective, governments of major exporters to Korea implement strategies to secure their own food supplies, and these policies interact within the global trade system, influencing food availability in Korea. Capturing these complex dynamics and idiosyncrasies within a single model is not only challenging but also limits our ability to effectively address the key questions we aim to answer.
